# High miR-24 expression is associated with risk of relapse and poor survival in acute leukemia

**DOI:** 10.3892/or.2015.3787

**Published:** 2015-02-06

**Authors:** JORGE ORGANISTA-NAVA, YAZMÍN GÓMEZ-GÓMEZ, BERENICE ILLADES-AGUIAR, LUZ DEL CARMEN ALARCÓN-ROMERO, MÓNICA VIRGINIA SAAVEDRA-HERRERA, ANA BERTHA RIVERA-RAMÍREZ, VÍCTOR HUGO GARZÓN-BARRIENTOS, MARCO ANTONIO LEYVA-VÁZQUEZ

**Affiliations:** 1 Institute of Cellular Physiology, National Autonomous University of Mexico (UNAM), University City, D.F., Mexico; 2 Research Department, State Cancer Institute, ‘Arturo Beltran Ortega’, Acapulco, Guerrero, Mexico; 3 Laboratory of Molecular Biomedicine, School of Chemical-Biological Sciences, Guerrero State University, Chilpancingo, Guerrero, Mexico; 4 Laboratory of Cytopathology, School of Chemical-Biological Sciences, Guerrero State University, Chilpancingo, Guerrero, Mexico

**Keywords:** miR-24, acute leukemia, acute myeloid leukemia, acute lymphoblastic leukemia, prognosis in acute leukemia

## Abstract

MicroRNAs (miRNAs) play an essential role in the development and progression of acute leukemia (AL). miR-24 promotes the survival of hematopoietic cells. However, little is known concerning the function of miR-24 in human AL. The aim of the present study was to investigate the clinical significance of miR-24 expression in AL. miR-24 expression in 147 patients with AL and 100 healthy individuals was measured by quantitative reverse transcriptase-polymerase chain reaction (RT-qPCR). The results showed that compared with the healthy individuals, the expression of miR-24 in AL patients was significantly higher (p<0.001). In addition, miR-24 was expressed at significantly higher levels in acute myeloid leukemia (AML) patients and at significantly lower levels in acute lymphoblastic leukemia (ALL) (p<0.001). More importantly, Kaplan-Meier analysis showed that AL patients with high miR-24 expression tended to have shorter overall survival (p<0.05). In the multivariate analysis stratified for known prognostic variables, miR-24 was identified as an independent prognostic marker. Our data indicated that miR-24 upregulation was associated with poor prognosis in AL. miR-24 was identified for the first time as an independent marker for predicting the clinical outcome of AL patients.

## Introduction

Acute leukemia (AL) is the most common cancer in childhood and is characterized by increased self-renewal of leukemia stem/progenitor cells, decreased cell death and a block in cell differentiation (1,2). In Mexico it has been observed that of the two types of AL, acute lymphoblastic leukemia (ALL) shows the highest frequency, accounting for 85% of the cases, while acute myeloid leukemia (AML) constitutes 15% (3).

MicroRNAs (miRNAs) are ~22 nt, non-coding RNA molecules that play a role in most cellular processes, including apoptosis, cell differentiation, proliferation and survival pathways (4). Furthermore, cancer-specific miRNA profiles associated with diagnosis, staging, progression, prognosis and response to treatment have been identified in many types of cancers (5). In leukemia, miRNA expression signatures associated with the cytogenetic and clinical outcome of adult CLL, AML and Hodgkin lymphoma were have been reported (6–8).

Recently, it was observed that miRNAs play a major regulatory role in normal hematopoietic differentiation, evidenced by the discovery of a small set of hematopoietic stem-progenitor cell (HSPC)-expressed miRNAs (HE-miRNAs) which post-transcriptionally regulate specific mRNAs involved in hematopoiesis (9–11).

miR-24 was found to be enriched in CD34^+^ HSPCs (9), and has a well-defined role as a regulator of normal erythropoiesis via targeting of human activin receptor type 1, ALK4 (12). Additionally, miR-24 is implicated in regulating apoptosis and cell proliferation. Reported targets of miR-24 include pro-apoptotic (FAF-1, caspase 9, Bim and Apaf-1) and cell cycle proteins (13–15) and it was observed that miR-24 promotes the survival of hematopoietic cells (16).

Previous studies have identified the processes in which miR-24 is involved in hematopoietic cell lines. However, the number of studies on the miR-24 expression features and functions in samples from pediatric patients with AL is relatively low. In the present study, we investigated the expression of miR-24 in clinical samples from children with AL, as well as healthy controls. Our primary aim was to investigate the differential expression of miR-24 in patients with AL and healthy individuals. Secondly, we determined if there was a significant association between miR-24 expression and patient survival, which could point to a potential role for miR-24 as a prognostic marker of AL.

## Materials and methods

### Study population

A case control study was carried out in the Pediatric Oncology Service of the State Cancer Institute (SCI) from the South of Mexico (Acapulco, Guerrero, Mexico), between September 2005 and July 2013. The cases consisted of 111 (ALL) and 36 (AML) patients diagnosed with AL, through bone marrow aspirate based on French-American-British morphological criteria of blast cells.

The diagnosis of ALL was further subclassified as T-lineage (CD3^+^, CD7^+^ plus CD2^+^ or CD5^+^ or both) or B-lineage (CD22^+^, CD19^+^, CD20^+^, CD79A^+^, HLA-DR^+^ and CD10^+^) (Becton-Dickinson Biosciences, Mountain View, CA, USA). The multiagent chemotherapeutic protocols used were 96091, 96092 or CIE-10:C9.1.0 of the Cancer Institute from Guerrero State and previously described (17,18).

Immunophenotypic studies to myeloblastic leukemia showed HLA-DR^+^, CD13^+^, CD19^−^, CD33^+^, CD45^+^, CD34^+^ and CD117^−^. Patients with AML were treated with cytarabine, mitoxantrone, daunorubicin and etoposide, according to the protocols of the Cancer Institute from Guerrero State (Tables I and II).

Complete remission was defined by <5% blast cells in the bone marrow and normalization of the peripheral blood counts at 4 weeks after starting induction therapy. Relapse was defined as the reappearance of >20% blast cells in the marrow, or the presence of localized leukemic infiltrates at any site after completion of induction chemotherapy (17,19). The worst outcome was defined as a lack of response to induction therapy, a relapse after achieving complete remission or death due to any cause (17,19). Overall survival (OS) was measured from the day of registration of the study until death from any cause, censored for patients known to be alive at the last contact. Risk classification was: standard risk, 1–10 years of age and presenting a white blood cell (WBC) count of <50,000/mm^3^; high-risk, <1 and >10 years of age; and a WBC count >50,000/mm^3^ (17,20). The controls were 100 apparently healthy individuals (4–10×10^3^ leukocytes/mm^3^) without a family history of leukemia. Subjects in both groups in the present study were 1–18 years of age, including both genders and were residents of the State of Guerrero, Mexico.

The bone marrow samples of the patients and blood samples of the healthy individuals used in the present study were part of the samples taken for clinical diagnostic tests in the hospital. No extra amount of samples was collected from the study subjects. Informed consent was obtained from all the individuals or their guardians, after a detailed briefing of the study aims. The present study and the informed consent procedure were approved by the Institutional Review Board of the Cancer Institute of the State of Guerrero, Mexico.

### Specimen collection and total RNA extraction

A bone marrow and/or a blood sample were taken from the 247 participants and placed in tubes with anticoagulant. Leukocytes were purified from the whole blood sample by a selective osmotic lysis of erythrocytes (21); total RNA was extracted using the TRIzol method (Invitrogen, Carlsbad, CA, USA) according to the manufacturer’s protocol and the quantity and concentration of RNA were spectrophotometrically assessed by measuring absorbance at A260/280.

### Detection of translocations by polymerase chain reaction

Total RNA (1 μg) was reverse transcribed into cDNA by priming with oligo(dT) and by the Superscript II First-Strand Synthesis System (Invitrogen) according to the manufacturer’s instructions. After the synthesis of cDNA, translocations were detected by the molecular biology technique of conventional PCR. Specific primers were used in the PCR reaction to amplify BCR-ABL, ETV6-RUNX1, AML1-ETO and CBFβ-MYH11 (22–26). The oligonucleotide sequences of these primers for each of these translocations are shown in Table III. Amplification was performed with a thermocycler Mastercycle ep gradient S (Eppendorf North America, Westbury, NY, USA).

For the amplification, cDNA (10 μg) was brought to a final volume of 50 ml with 1X PCR buffer, 0.2 mM of each dNTP, 1.5 mM MgCl_2_, 1 U *Taq* polymerase (all from Invitrogen) and 0.4 μM of each primer. For all the translocations and the constitutive gene, the conditions for amplification are shown in Table III using previously established protocols (22–26). The amplification products were subjected to electrophoresis on a 2.5% agarose gel, stained with ethidium bromide and viewed under an UV transilluminator. The amplification products could be discriminated by molecular size using a molecular weight marker (100 bp; Invitrogen).

### Quantification of miRNAs using real-time PCR

To detect the levels of miR-24, 1–10 ng of total RNA was reverse transcribed to cmiRNA with specific RT primer using TaqMan^®^ MicroRNA Reverse Transcription kit, and stem-loop real-time PCR was used to detect miR-24 level by the TaqMan^®^ MicroRNA assays (000402) (both from Applied Biosystems, Foster City, CA, USA). The PCR cycles were as follows: 94°C for 5 min, followed by 40 cycles of 94°C for 30 sec, 60°C for 30 sec and 72°C for 30 sec. Real-time reverse transcription polymerase chain reactions were performed in an Applied Biosystems 7500 Detection System (Applied Biosystems).

The expression of miR-24 was determined from the threshold cycle (Ct), and the relative expression levels were calculated by the 2^−ΔΔCt^ method. The Ct values were normalized with reference to the expression of RNU6B (001093; Applied Biosystems).

### Statistical analysis

Continuous data are presented as the means ± standard deviation (SD) or median, 25th and 75th interquartiles. Categorical data were compared by Chi-square or Fisher’s exact tests. One-way analysis of variance (ANOVA) was used to compare differences among the miR-24 levels between groups, and results are presented as mean ± SD. Univariate logistic regression analysis for the association with the risk of relapse to AL were tested first for miR-24 expression, gender and other clinical characteristics, and those factors were included into a second multivariate logistic analysis. The log-rank test and Kaplan-Meier curves were used to analyze the effect of the miR-24 expression, gender, risk of relapse and risk classification (standard- and high-risk) on OS. p<0.05 was considered to indicate a statistically significant result. All statistical analyses were performed using SPSS software, version 20.0 (SPSS, Inc., Chicago, IL, USA), GraphPad Prism software (version 5.0; GraphPad Software, Inc., USA) and STATA software, version 9.2 (StataCorp, College Station, TX, USA).

## Results

### General characteristics of the children with AL

We studied 111 children with ALL with a mean age of 7.73±4.91 (mean ± SD) years and a median leukocyte count at diagnosis of 19,700 leukocytes/mm^3^. The predominant gender was male with 63.06% while there were 36.94% female patients. These children (63.96%) had a relapse of ALL at some time during their treatment. According to risk by age and leukocytes at diagnosis 43.24% of the children were in the age group of 1–9 years, while 56.76% of the patients were <1 and >9 years of age at the time of the initial diagnosis.

Of the 111 cases with ALL examined by immunophenotype, B-lineage was the most frequently found (83.78%). The majority (83.78%) was cytomorphologically diagnosed as L1 (Table IV). Seven (6.31%) cases of ALL presented with the BCR-ABL rearrangement; 1 case (0.90%) the ETV6-RUNX1 rearrangement; while, 64 (57.66%) showed none of the genetic rearrangements under study (BCR-ABL or ETV6-RUNX1 rearrangements). Thirty nine of the 111 patients with ALL were not considered for rearrangement analysis since analysis was not possible (Table IV).

Likewise, we also included 36 children with AML, who had a mean age of 8.102±4.79 years, with a median of 34, 550 leukocytes/mm^3^ at diagnosis. There were 22 (61.11%) males and 14 (38.89%) females. Twelve patients (33.33%) were in the age group of 1–9 years. Twenty-four patients (66.67%) were <1 and >9 years of age at the time of the initial diagnosis. Nineteen (52.77%) of the patients with AML had a relapse at some time during their treatment (Table IV).

TheFABsubtypesobservedinthepresentstudywereM0–M3, with a preponderance of acute myeloblastic leukemia without maturation (FAB-M1, 38.89%), followed by minimally differentiated acute myeloid leukemia (FAB-M0, 27.77%). Adequate rearrangement analyses were obtained for 25 (69.44%) of the patients with AML. Three (8.33%) cases of AML presented with the AML1-ETO rearrangement; while, 22 (61.11%) showed none of the genetic rearrangements under study (AML1-ETO or CBFB-MYH11 rearrangements). Eleven of the 36 patients with AML studied were not considered for rearrangement analysis since analysis was not possible (Table IV).

### General characteristics of the healthy children

One-hundred healthy individuals (controls) were apparently included. In this group, the age range was 1–18 years (mean ± SD, 10.21±5.53 years), and the leukocyte count was normal (4–10×10^3^ leukocytes/mm^3^; median 8,000). In this group, 53 healthy individuals (53.00%) were male and 47 (47.00%) were female (Table IV). A significant difference could be observed in the age and the leukocyte count at diagnosis between the patients with AL and healthy individuals (Table IV).

### miR-24 was differentially expressed in AML and ALL

**Figure 1:**
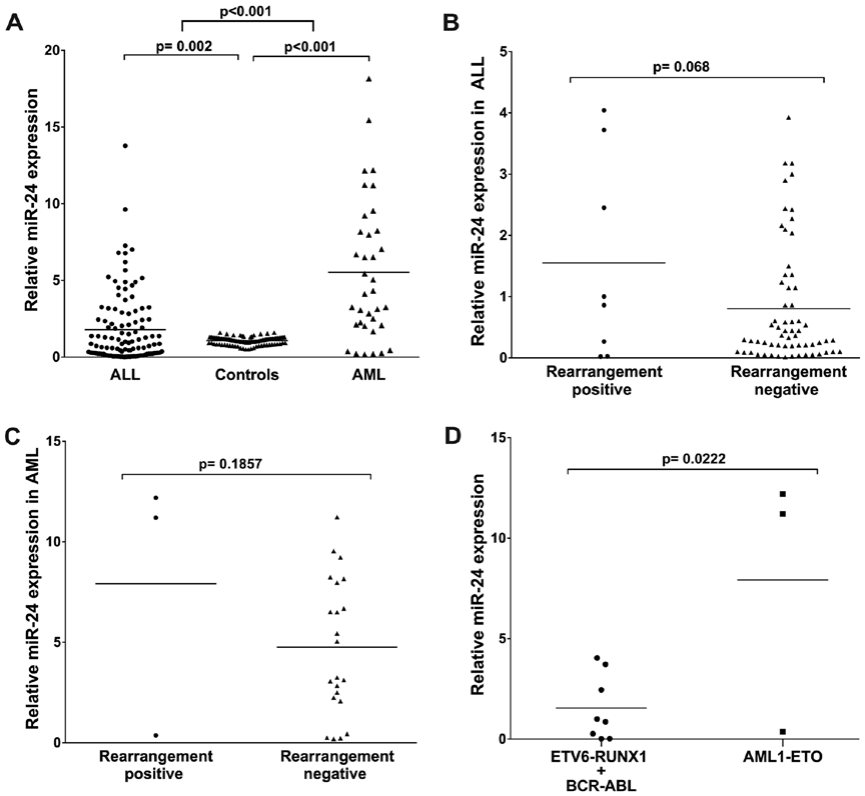
miR-24 expression in AL patients and healthy individuals. (A) The expression level of miR-24 in AML patients was significantly higher [median (25–75 percentiles), 4.22 (2.08–8.22); p<0.001]. miR-24 in ALL patients was significantly lower [median (25–75 percentiles), 0.84 (0.21–2.54); p<0.001]. (B) Levels of miR-24 expression in ALL patients with/without rearrangement were not significant (p=0.068). (C) Expression level of miR-24 in AML patients with/without rearrangement was not significant (p=0.185). (D) The expression level of miR-24 in AML1-ETO rearrangement-positive AML patients were significantly higher (median, 7.92; p=0.022). While miR-24 in ETV6-RUNX1/BCR-ABL rearrangement-positive ALL patients was significantly lower (median, 1.55; p=0.022). AL, acute leukemia; ALL, acute lymphoblastic leukemia; AML, acute myeloblastic leukemia.

To identify whether miR-24 was differentially expressed between the ALL and AML samples, we examined miR-24 levels in samples of the AL patients and the healthy individuals. The RNAs isolated were subjected to TaqMan RT-PCR analysis. The assays showed that the miR-24 levels were significantly higher (4.22 median, p<0.001) in the AML patients as compared with the healthy individuals. In turn, miR-24 in the ALL patients was significantly low (0.84 median, p=0.002). miR-24 expression in the AML patients was much higher than that in the ALL patients (p<0.001) (Table IV and Fig. 1A). These results suggest that upregulation of miR-24 expression may play a role in the progression of AML.

### miR-24 expression in AL patients with/without rearrangement

We further quantitatively detected miR-24 expression in 111 cases of ALL and 36 cases of AML divided into two subtype groups: rearrangement-positive (ALL, 8; AML, 3) and rearrangement-negative (ALL, 64; AML, 22). The analysis showed no statistically significant difference between the positive and negative rearrangement patients (p=0.068 for ALL and p=0.185 for AML), (Fig. 1B and C).

Then, we compared miR-24 expression in patients according to ETV6-RUNX1/BCR-ABL vs. AML1-ETO rearrangement positivity and observed that miR-24 was significantly higher in the AML1-ETO-positive patients (p=0.022); the mean was 1.55-fold (ETV6-RUNX1/BCR-ABL) vs. 7.92-fold (AML1-ETO) (Fig. 1D).

### Risk of relapse based on the miR-24 expression and other risk factors

To evaluate the correlation between miR-24 expression and the risk of relapse to ALL, patients were divided into groups with downregulation and upregulation of miR-24 expression. The 75th percentile expression level of miR-24 (2.54-fold) was used as a cut-off point to divide all 111 patients with ALL into 2 groups. Those who expressed miR-24 at levels less than the cut-off value were assigned to the downregulation group (n=61), and those with expression above the cut-off value were assigned to the upregulation group (n=50).

In a logistic regression analysis, an association was observed between miR-24 expression and the risk of relapse of ALL (p<0.05). It was observed that those patients with upexpression of miR-24, showed a significant increase in the risk of relapse to ALL (OR=2.51, 95% CI 1.10–5.72, p=0.028) compared to those patients who had downexpression of miR-24 expression (Table V). We also observed that individuals <1 and >10 years of age with >50,000 leukocytes/mm^3^ (high-risk) had a 5.46 fold (95% CI 2.34–12.77, p≤0.001) increased risk to have relapsed compared to individuals between 1–10 years with <50,000 leukocytes/mm^3^ (low-risk) (Table V).

Similarly to what was carried out with patients with ALL, the patients with AML were divided in subgroups according to downexpression and upexpression [< or > 75th percentile expression level of miR-24 (8.22-fold)]: downexpression group (n=18) and upexpression group (n=18). We also examined the relationship between miR-24 expression levels and the risk of relapse to AML. We observed a significant correlation between miR-24 expression levels and risk of relapse (OR=7.00, 95% CI 1.59–30.79, p=0.010), (Table VI). These data suggest that upregulation of miR-24 expression may have an important role in the relapse of the disease.

### Expression of miR-24 is associated with unfavorable prognosis in AL patients

**Figure 2:**
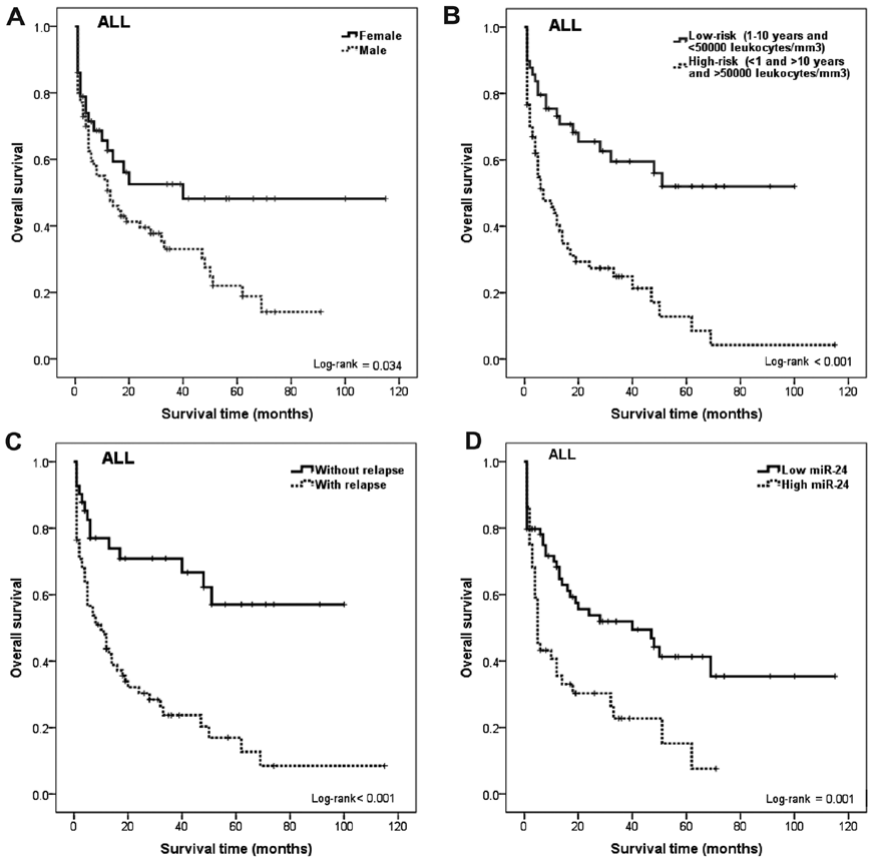
Kaplan-Meier curves for overall survival time influence of gender, risk by age and leukocyte count at diagnosis, relapse and miR-24 expression of ALL patients. (A) OS in females and males with ALL. Significantly shorter OS for males than female patients was noted (p=0.034). (B) OS between patients with low risk (1–10 years of age and <50,000 leukocytes/mm^3^) and high risk (<1 and >10 years of age and >50,000 leukocytes/mm^3^). Significantly longer OS for patients with a low risk than patients with high-risk was noted (p<0.001). (C) OS between patients with and without relapse. Significantly shorter OS for patients with relapse than patients without relapse was noted (p<0.001). (D) OS was significantly shorter for patients with high miR-24 expression than for those with low miR-24 expression (p=0.001). ALL, acute lymphoblastic leukemia; OS, overall survival.

**Figure 3:**
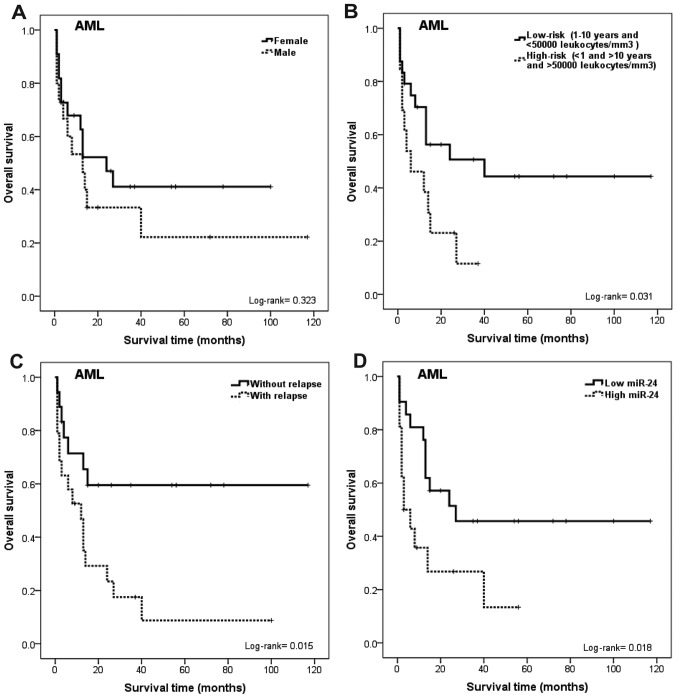
Kaplan-Meier curves for overall survival time considering the influence of gender, risk by age and leukocyte count at diagnosis, relapse and miR-24 expression of AML patients. (A) OS in females and males with AML. Shorter OS for male than female patients was noted (although it was not statistically significant, p=0.323). (B) OS between patients with low-risk (1–10 years of age and <50,000 leukocytes/mm^3^) and high-risk (<1 and >10 years of age and >50,000 leukocytes/mm^3^). Significantly longer OS for patients with a low-risk than patients with high-risk (p=0.031). (C) OS between patients with and without relapse. Significantly shorter OS for patients with relapse than patients without relapse was noted (p=0.015). (D) OS significantly shorter for patients with high miR-24 expression than for those with low miR-24 expression (p=0.018). AML, acute myeloblastic leukemia; OS, overall survival.

The relationship between survival and gender was also calculated. We observed that males had a poorer OS compared to females (log-rank test; p=0.034 for ALL, Fig. 2A). In AML the Kaplan-Meier survival curves showed no significant association between gender and survival, although a reduction in survival among men and women was observed (log-rank test; p=0.323; Fig. 3A).

Likewise, individuals <1 and >10 years of age with >50,000 leukocytes/mm^3^ (high-risk) had a poor survival rate compared with those patients between 1–10 years of age with <50,000 leukocytes/mm^3^ (Fig. 2B; log-rank test; p<0.001 for ALL and Fig. 3B; log-rank test; p=0.031 for AML). A different rate of OS was evident between the individuals with and without relapse of AL (Fig. 2C; log-rank test; p<0.001 for ALL and Fig. 3C; log-rank test; p=0.015 for AML).

The association between miR-24 expression and survival of AL patients was investigated. We observed that ALL patients with high miR-24 expression tend to have shorter OS than those with low miR-24 (Fig. 2D; log-rank test; p=0.001 for ALL and Fig. 3D; log-rank test; p=0.018 for AML). During the follow-up period, 69 of the 111 patients with ALL (62.16%) and 23 of the 36 patients with AML (63.89%) had died (Table IV). In addition, the univariate and multivariate analyses performed showed that miR-24 expression is an independent prognostic factor for AL (Tables V and VI).

## Discussion

Acute leukemias (ALs) are the most frequent type of childhood cancers (2). In spite of the development of advanced therapeutic strategies, the prognosis of patients with this type of cancer varies significantly and is hard to predict; therefore knowledge of the prognosis is vital since some patients with AL have different responses to the same therapy (27). Therefore, it is critical to identify biomarkers for the early identification of patients with a high-risk of treatment failures, in order to modify therapeutic methods for improving the overall survival (OS) of patients with AL.

miR-24 is a tumor-suppressor among the miRNAs that are consistently upregulated during terminal differentiation. miR-24 is expressed in a cyclical manner and takes part in maintaining and regulating proper cell cycle progression and apoptosis (13,15). Overexpression of miR-24 in liver, gastric, prostate and cervical cancer cell lines was found to protects these cells from apoptosis whereas knockdown of miR-24 turns differentiated cells into a proliferation state and sensitizes them to apoptosis (13,15). Yet the role of miR-24 in AL samples is poorly understood. We investigated the expression of miR-24 in samples of AL patients and detected its relationships with clinical parameters.

In the present study, we observed that miR-24 expression was significantly increased in both AML and ALL patients (p<0.001). This phenomenon was consistent with previous publications, where it was noted that miR-24 promotes the survival of hematopoietic progenitors (16,28).

A previous study has shown miR-24 to have an increased expression in AML and a decreased expression in ALL (28). For a more comprehensive insight into the clinical value of miR-24, in the present study we performed a logistic regression analysis to investigate its association with the clinical features of AL patients. Our data proved that miR-24 expression was significantly higher in AL patients compared with that in apparently healthy individuals (p<0.001). We observed a statistically significant association between the expression of miR-24 and the risk of AL (OR=2.51, 95% CI 1.10–5.72, p=0.028 for ALL and OR=7.00, 95% CI 1.59–30.79, p=0.010 for AML). In addition, miR-24 expression, was associated with risk of relapse of leukemia (p<0.05). This suggests that the regulation of miR-24 expression, and the high association with the risk of relapse (p<0.05) may be a factor that led to >50% of deaths in the patients with AL included in the present study.

Moreover, it was reported that miRNA expression is correlated with cytogenetic and molecular subtypes of AL [i.e., with t(8;21), t(15;17), inv(16), NPM1 and CEBPA mutations] (8). Notably, two from our cases with t(8;21) rearrangement presented high expression of miR-24 when compared with others rearrangements (ETV6-RUNX1/BCR-ABL), which is similar to previous findings (8,28–30).

In many studies, age, gender and white cell count at diagnosis have been shown to be a consistently strong prognostic factors in childhood AL. Patients <1 year of age have higher relapse rates compared with others ages as reported in various studies; this feature also retained its significance in this study. Patients in the age group of 1–10 years (low-risk) had the best prognosis, whereas patients <1 and >9 years of age (high-risk) showed the worst prognosis (OR=5.46, 95% CI 2.34–12.77, p≤0.001 for ALL and OR=5.57, 95% CI 1.29–23.93, p=0.021 for AML), which agrees with previous studies (17,31–33). The relationship between white cell count at diagnosis (WBC) and prognosis has been firmly established in many studies of childhood AL (17,30–33) and confirmed in the present study. WBC >50,000 leukocytes/mm^3^ (high-risk) has been shown to be an adverse risk factor in childhood AL. This value has been proposed as the value by which to define patients with a poor prognosis by the National Cancer Institute sponsored workshop (20).

In the multivariate analysis, for patients with miR-24 expression, OR estimates retained their significance (p<0.05) in the presence of other prognostic factors, which also influenced AL outcome (age, gender and risk by age and leukocytes at diagnosis), which suggests that miR-24 is an independent prognostic marker for AL. More importantly, we proved that miR-24 expression was significantly associated with OS of patients with AL. In support of this, Kaplan-Meier analysis of OS showed that patients with high miR-24 expression tended to have a significantly shorter OS compared with patients with low expression (log-rank p<0.05), indicating that high miR-24 expression is a marker of poor prognosis for patients with AL. Thus, miR-24 could be used as molecular prognostic marker in addition to known prognostic indicators, in order to identify patients who are more likely to have a higher risk of death, thus, should receive more aggressive treatment.

In conclusion, our data indicated that miR-24 upregulation was associated with poor prognosis in AL. miR-24 was identified for the first time as an independent marker for predicting the clinical outcome of AL patients. Nevertheless, our data generate novel hypotheses regarding the role of miR-24 expression in the risk and relapse of AL and an impact on survival of AL patients, which will have to be confirmed in independent studies.

## Figures and Tables

**Table I:** Acute myeloid leukemia chemotherapy regimens.

Regimen		Dosing
		High-risk patients
Induction therapy	Cycle 1	
Cytarabine	Days 1–7:	Cytarabine 100 mg/m^2^/day continuous intravenous (IV) infusion for 2 h
Daunorubicin	Days 1, 3 and 5:	Daunorubicin 30 mg/m^2^/day continuous IV infusion for 1 h
Etoposide	Days 1–5:	Etoposide 100 mg/m^2^/day IV for 3 h
Mitoxantrone	Day 2:	Intrathecal (IT) chemotherapy (Table II)
	Cycle 2	
	Days 1–7:	Cytarabine 100 mg/m^2^/day continuous IV infusion for 2 h
	Days 1, 3. 5:	Daunorubicin 30 mg/m^2^/day continuous IV infusion for 1 h
	Days 1–5:	Etoposide 100 mg/m^2^/day continuous IV infusion for 3 h
	Day 2:	IT chemotherapy (Table II)
	Cycle 3	
	Days 1–5:	Cytarabine 100 mg/m^2^/day continuous IV infusion for 2 h
	Days 1–3:	Mitoxantrone 10 mg/m^2^/day continuous IV infusion for 30 min
	Day 2:	Intrathecal (IT) chemotherapy (Table II)
Maintenance therapy	Cycle 1	
Cytarabine	Days 1–3:	Cytarabine 1 g/m^2^/day continuous IV infusion for 2 h
Daunorubicin	Days 1–3:	Mitoxantrone 10 mg/m^2^/day continuous IV infusion for 30 min
Etoposide	Day 2:	Intrathecal (IT) chemotherapy (Table II)
Mitoxantrone	Cycle 2	
	Days 1–3:	Cytarabine 2 g/m^2^/day continuous IV infusion for 2 h
	Days 1–4:	Etoposide 100 mg/m^2^/day continuous IV infusion for 1 h
	Day 2:	Intrathecal (IT) chemotherapy (Table II)
	Cycle 3	
	Days 1–3:	Cytarabine 3 g/m^2^/day continuous IV infusion for 2 h
	Cycle 4	
	Days 1–3:	Cytarabine 2 g/m^2^/day continuous IV infusion for 2 h
	Days 1–4:	Etoposide 100 mg/m^2^/day continuous IV infusion for 1 h
	Day 2:	Intrathecal (IT) chemotherapy (Table II)
	Low-risk patients	
Induction therapy	Cycle 1	
Cytarabine	Days 1–7:	Cytarabine 100 mg/m^2^/day continuous intravenous (IV) infusion for 2 h
Daunorubicin	Days 1, 3 and 5:	Daunorubicin 30 mg/m^2^/day continuous IV infusion for 1 h
Etoposide	Days 1–5:	Etoposide 100 mg/m^2^/day IV for 3 h
	Day 2:	Intrathecal (IT) chemotherapy (Table II)
	Cycle 2	
	Days 1–7:	Cytarabine 100 mg/m^2^/day continuous IV infusion for 2 h
	Days 1, 3 and 5:	Daunorubicin 30 mg/m^2^/day continuous IV infusion for 1 h
	Days 1–5:	Etoposide 100 mg/m^2^/day continuous IV infusion for 3 h
Maintenance therapy	Cycle 1	
Cytarabine	Days 1–3:	Cytarabine 1 g/m^2^/day continuous IV infusion for 2 h
Daunorubicin	Days 1–3:	Daunorubicin 25 mg/m^2^/day continuous IV infusion for 1 h
Etoposide	Cycle 2	
	Days 1–3:	Cytarabine 1 g/m^2^/day continuous IV infusion for 2 h
	Days 1–4:	Etoposide 100 mg/m^2^/day continuous IV infusion for 1 h
	Cycle 3	
	Days 1–3:	Cytarabine 2 g/m^2^/day continuous IV infusion for 2 h
	Cycle 4	
	Days 1–3:	Cytarabine 1 g/m^2^/day continuous IV infusion for 2 h
	Days 1–4:	Etoposide 100 mg/m^2^/day continuous IV infusion for 1 h

**Table II:** Acute myeloid leukemia intrathecal (IT) chemotherapy.

Age (years)	Methotrexate (mg)	Hydrocortisone (mg)	ARA-C (mg)	Volume (ml)
<2	8	16	24	8
2	10	20	30	10
>3	12	24	36	12

**Table III:** Oligonucleotide sequences of the primers used in this study.

Genetic fusion	Sense strand	Antisense strand	Size (bp)
BCR-ABL			
Subtype b3a2	5′-TCGTGTGTGAAACTCCAGAC-3′	5′-CCATTCCCCATTGTGATTAT-3′	349
Subtype b2a2	5′-TCGTGTGTGAAACTCCAGAC-3′	5′-CCATTCCCCATTGTGATTAT-3′	274
Subtype e1a2	5′-ACTGCCCGGTTGTCGTGT-3′	5′-CCATTCCCCATTGTGATTAT-3′	317
Internal control ABL	5′-TAGCATCTGACTTTGAGCCT-3′	5′-CCATTCCCCATTGTGATTAT-3′	200
ETV6-RUNX1			
Subtype 1s1	5′-AGCCCCATCATGCACCCTCTGATCC-3′	5′-GTGGTCGGCCAGCACCTCCACC-3′	271
Subtype 1s2	5′-GCAGAATTCCACTCCGTGGATTTCAAACAGTCC-3′	5′-AACGCCTCGCTCATCTTGCCTGGGCTC-3′	232
Internal control BL1	5′-GAGGGAAAAGCTTCACTCTG-3′	5′-GCCGCAGCTGCTCCAGTTCA-3′	200
AML1-ETO			
AML1-ETO	5′-GAGGGAAAAGCTTCACTCTG-3′	5′-GCGAACTCTTTCTCCTATC-3′	467
Internal control AML	5′-GAGGGAAAAGCTTCACTCTG-3′	5′-GCCGCAGCTGCTCCAGTTCA-3′	192
CBFB-MYH11			
Subtype A	5′-AGCTGCGTCTTCATCTCCTC-3′	5′-CTGGATGGTATGGGCTGTCT-3′	227
**S**ubtype B	5′-AGCTGCGTCTTCATCTCCTC-3′	5′-CTGGATGGTATGGGCTGTCT-3′	241
Subtype B	5′-GTCTGTGTTATCTGGAAAGGCTG-3′	5′-CGTACTGCTGGGTGAGGTTCT-3′	620
Subtype C	5′-GTCTGTGTTATCTGGAAAGGCTG-3′	5′-CGTACTGCTGGGTGAGGTTCT-3′	568
Subtype D	5′-GTCTGTGTTATCTGGAAAGGCTG-3′	5′-CGTACTGCTGGGTGAGGTTCT-3′	775
Internal control CBFβ	5′-CTGGATGGTATGGGCTGTCT-3′	5′-TAGGGTCTTGTTGTCTTCTTGC-3′	230

**Table IV:** General characteristics and clinical data of the AL patients and healthy individuals.

Variables	ALL 111 (75.51)	AML 36 (24.49)	Healthy individuals 100 (100)	P-value^b^
Age (years, mean ± SD)	7.73±4.91	8.02±4.79	10.21±5.53	0.002^d^
No. of leukocytes/mm^3^	19,700 (4,700–42,900)^a^	34,550 (9,350–68,000)^a^	8,000 (7,000–9,000)^a^	<0.001^d^
Gender				
Female	41 (36.94)	14 (38.89)	47 (47.00)	0.317
Male	70 (63.06)	22 (61.11)	53 (53.00)	
Status of participants				
Alive	42 (37.84)	13 (36.11)	100 (100.00)	1.00^c^
Deceased	69 (62.16)	23 (63.89)	-	
Risk by age and leukocytes at diagnosis				
Low-risk (1–10 years and <50,000 leukocytes/mm^3^)	48 (43.24)	12 (33.33)	-	1.00^c^
High-risk (<1 and >10 years and >50,000 leukocytes/mm^3^)	63 (56.76)	24 (66.67)	-	
Relapse during treatment				
No	40 (36.04)	17 (47.22)	-	
Yes	71 (63.96)	19 (52.77)	-	0.244^c^
Immunophenotype				
B-lineage	93 (83.78)	-	-	-
T-lineage	11 (9.91)	-	-	
B/T-lineage	7 (6.31)	-	-	
FAB classification				
L1	93 (83.78)	-	-	-
L2	18 (16.22)	-	-	
M0	-	10 (27.77)	-	
M1	-	14 (38.89)	-	-
M2	-	6 (16.67)	-	
M3	-	6 (16.67)	-	
Chromosomal translocations				
ETV6-RUNX1 [t(12;21)]	1 (0.90)	-	-	
BCR-ABL [t(9;22)]	7 (6.31)	-	-	
AML1-ETO [t(8;21)]	-	3 (8.33)	-	
CBFB-MYH11 [inv(16)]	-	0 (0.00)	-	-
Negative	64 (57.66)	22 (61.11)	-	
Not determined	39 (35.13)	11 (30.56)	-	
miR-24 levels	0.84 (0.21–2.54)^a^	4.22 (2.08–8.22)^a^	1.25 (1.09–1.61)^a^	<0.001^d^

Data are expressed as n (%) unless indicated otherwise;

a median (percentiles 25–75).

b Obtained by the Chi-square test.

c p-value obtained between patients with ALL and AML.

d Significant p<0.05.

AL, acute leukemia; ALL, acute lymphoblastic leukemia AML, acute myeloid leukemia.

**Table V:** Association of miR-24 expression and clinical features with the risk of relapse to ALL.

	Without relapse n (%)	With relapse n (%)	P-value	Univariate analysis			Multivariate analysis		
	
				OR	CI 95%	P-value^a^	OR	CI 95%	P-value^b^
Gender									
Female	16 (41.03)	25 (34.72)	0.542	1.00					
Male	23 (58.97)	47 (65.28)		1.31	0.59–2.91	0.512			
Immunophenotype									
B-lineage	36 (92.31)	57 (79.17)		1.00					
T-lineage	2 (5.13)	9 (12.50)	0.198	2.84	0.58–13.90	0.197			
B/T-lineage	1 (2.56)	6 (8.33)		3.78	0.44–32.78	0.226			
B-lineage	36 (92.31)	57 (79.17)		1.00					
T-lineage + B/T-lineage	3 (7.69)	15 (20.83)	0.105	3.15	0.85–11.67	0.085			
FAB classification									
ALL									
L1	31 (79.49)	62 (86.11)		1.60	0.57–4.46	0.369			
L2	8 (20.51)	10 (13.89)	0.423	1.00					
Risk by age and leukocytes at diagnosis									
Low-risk (1–10 years and <50,000 leukocytes/mm^3^)	27 (69.23)	21 (29.17)	<0.001^c^	1.00					
High-risk (<1 and >10 years and >50,000 leukocytes/mm^3^)	12 (30.77)	51 (70.83)		5.46	2.34–12.77	<0.001^c^	5.20	2.19–12.32	<0.001^c^
miR-24 levels									
Downregulated	27 (69.23)	34 (47.22)	0.030^c^	1.00					
Upregulated	12 (30.77)	38 (52.78)		2.51	1.10–5.72	0.028^c^	2.27	1. 94–5.51	0.020^c^

OR, odds ratio; CI, confidence interval;

a p-value was obtained by logistic regression analysis, taking reference to female, B-lineage, L2, 1–10 years, <50,000 leukocytes/mm^3^ (low-risk) and downregulated levels of miR-24.

b p-value was obtained by multivariate logistic regression analysis.

c Significant p<0.05, ALL, acute lymphoblastic leukemia.

**Table VI:** Association of the miR-24 expression and clinical features with risk of relapse to AML.

	Without relapse n (%)	With relapse n (%)	P-value	Univariate analysis			Multivariate analysis		
	
				OR	CI 95%	P-value^a^	OR	CI 95%	P-value^b^
Gender									
Female	7 (43.75)	7 (35.00)	0.734	1.00					
Male	9 (56.25)	13 (65.00)		1.44	0.37–5.57	0.593			
FAB classification									
M0	5 (31.25)	5 (25.00)		0.73	0.17–3.17	0.678			
M1	7 (50.00)	5 (30.00)	0.460	0.42	0.10–1.76	0.240			
M2	2 (6.25)	6 (15.00)		3.00	0.51–17.49	0.222			
M3	2 (12.50)	4 (20.00)		1.00					
Risk by age and leukocytes at diagnosis									
Low-risk (1–10 years and <50,000 leukocytes/mm^3^)	12 (75.00)	7 (35.00)	0.023^c^	1.00					
High-risk (<1 and >10 years and >50,000 leukocytes/mm^3^)	4 (25.00)	13 (65.00)		5.57	1.29–23.93	0.021^c^	8.38	1.39–50.56	0.020^c^
miR-24 levels									
Downregulated	12 (75.00)	6 (30.00)	0.018^c^	1.00					
Upregulated	4 (25.00)	14 (70.00)		7.00	1.59–30.79	0.010^c^	10.20	1.71–60.87	0.011^c^

OR, odds ratio; CI, confidence interval;

a p-value was obtained by logistic regression analysis, taking reference to female, M3, 1–10 years, <50,000 leukocytes/mm^3^ (low-risk) and downregulated levels of miR-24.

b p-value was obtained by multivariate logistic regression analysis.

c Significant p<0.05.

AML, acute myeloblastic leukemia.
